# Complete Genome Sequence of the Solvent Producer *Clostridium saccharoperbutylacetonicum* Strain DSM 14923

**DOI:** 10.1128/genomeA.01056-14

**Published:** 2014-10-16

**Authors:** Anja Poehlein, Preben Krabben, Peter Dürre, Rolf Daniel

**Affiliations:** a Genomic and Applied Microbiology and Göttingen Genomics Laboratory, Georg-August University, Göttingen, Germany; b Green Biologics Ltd., Abingdon, Oxfordshire, United Kingdom; c Institut für Mikrobiologie und Biotechnologie, Universität Ulm, Ulm, Germany

## Abstract

*Clostridium saccharoperbutylacetonicum* strain DSM 14923 is known as a butanol-producing bacterium. Various organic compounds such as glucose, fructose, sucrose, mannose, and cellobiose are fermented. The genome consists of one chromosome and one circular megaplasmid. *C. saccharoperbutylacetonicum* was used in industrial fermentation processes to produce the solvents acetone, butanol, and ethanol.

## GENOME ANNOUNCEMENT

*Clostridium saccharoperbutylacetonicum* is an anaerobic, spore-forming, Gram-positive bacterium that produces solvents and hydrogen. The strain was first cultured from soil by Hongo et al. in 1959 as strain 97 (1, 2) and was subsequently used by the Sanraku Distillers Company for butanol production in the early 1960s, until production was stopped due to phage issues (3). Here, we report the closed and fully annotated genome sequence of *C. saccharoperbutylacetonicum* strain DSM 14923. This strain is also designated N1-4 (HMT). Another genome sequence of this organism is publicly available, but it is a draft sequence with 210 contigs (4).

The MasterPure complete DNA purification kit (Epicentre, Madison, WI, USA) was used to isolate chromosomal DNA of *C. saccharoperbutylacetonicum* DSM 14923. For whole-genome sequencing the Genome Analyzer II (Illumina, San Diego, CA, USA) and the 454 GS-FLX Titanium XL pyrosequencing system (Titanium Chemistry, Roche Life Science, Mannheim, Germany) were used. Preparation of shotgun libraries was performed according to the manufacturers’ protocols and resulted in 3,087,596 paired-end Illumina reads and 402,533 pyrosequencing reads. Initial hybrid *de novo* assembly using the MIRA software (5) resulted in 327 contigs and an average coverage of 58.29×. The remaining gaps were closed by PCR-based techniques and Sanger sequencing using BigDye 3.0 chemistry and an ABI3730XL capillary sequencer (Applied Biosystems, Life Technologies GmbH, Darmstadt, Germany). For this purpose, the Gap4 (version 4.11) software of the Staden package was employed (6). The complete genome of *C. saccharoperbutylacetonicum* DSM14923 comprises two replicons, a chromosome (6.53 Mb), and a circular megaplasmid (136 kb) with an overall GC content of 29.54%. This represents the highest genome size of all completely sequenced clostridial genomes. Automatic gene prediction was performed using YACOP and GLIMMER software (7). Identification of rRNA and tRNA genes was done with RNAmmer (8) and tRNAscan (9), respectively. The IMG/ER (Integrated Microbial Genomes/Expert Review) system (10, 11) was used for automatic annotation, which was subsequently manually curated by using the Swiss-Prot, TrEMBL, and InterPro databases (12). We identified 11 rRNA operons, 70 tRNA genes, 4,287 protein-encoding genes with function prediction, 1,534 genes coding for hypothetical proteins, and 11 pseudogenes. In contrast to *C. acetobutylicum* ATCC 824 (13), the *sol* operon of *C. saccharoperbutylacetonicum* is located on the chromosome and not on the megaplasmid. It consists of aldehyde dehydrogenase (*ald*), CoA transferase (*ctfAB*) and acetoacetate decarboxylase (*adc*). The *sol* operon of *C. saccharoperbutylacetonicum* shows the same arrangement as that of *C. beijerinckii* NCIMB8052 (14, 15) and *C. saccharobutylicum* (16) but differs from that of *C. acetobutylicum* ATCC 824, in which the *ald* gene is replaced by an alcohol/aldehyde dehydrogenase-encoding gene (*adhE*) and *adc* forms a separate operon. Based on a substrate preference for butyraldehyde, the aldehyde dehydrogenase of *C. saccharoperbutylacetonicum* was described as butyraldehyde dehydrogenase (17). Genes encoding acetyl-CoA acetyltransferase, crotonase, butyryl-CoA dehydrogenase, phosphate butyltransferase, butyrate kinase, phosphate acetyltransferase, acetate kinase, and several alcohol dehydrogenases, key enzymes for solvent production, were identified.

### Nucleotide sequence accession numbers.

The genome sequence was deposited in GenBank under accession numbers CP004121 (chromosome) and CP004122 (megaplasmid).
